# Circulating miR-583 and miR-663 Refer to ZHENG Differentiation in Chronic Hepatitis B

**DOI:** 10.1155/2013/751341

**Published:** 2013-03-10

**Authors:** Hui Zhang, Yan Guan, Yi-Yu Lu, Yi-Yang Hu, Shuang Huang, Shi-Bing Su

**Affiliations:** ^1^Research Center for Traditional Chinese Medicine Complexity System, Shanghai University of Traditional Chinese Medicine, 1200 Cailun Road, Pudong, Shanghai 201203, China; ^2^Institute of Liver Diseases, Shanghai Shuguang Hospital, Key Laboratory of Liver and Kidney Diseases of Ministry of Education, Shanghai University of Traditional Chinese Medicine, 528 Zhangheng Road, Pudong, Shanghai 201203, China; ^3^Department of Biochemistry and Molecular Biology, Georgia Regents University, Augusta, GA 30912, USA

## Abstract

Traditional Chinese medicine (TCM) ZHENG as the key pathological principle is to understand the human homeostasis and guide TCM treatment. Here, circulating microRNAs (miRNAs) were utilized to differentiate between ZHENGs including liver-gallbladder dampness-heat syndrome (LGDHS) and liver-kidney yin deficiency syndrome (LKYDS) in chronic hepatitis B (CHB). Sera samples of CHB patients with LGDHS (*n* = 35), LKYDS (*n* = 24), and healthy controls (Ctrls, *n* = 21) were analyzed by microarray and real-time RT-PCR. Receiver-operator characteristic (ROC) curves were established to evaluate the levels of serum miRNA for discriminating LGDHS and LKYDS. The target genes of miRNAs were predicted by TargetScan. Gene Ontology (GO) and pathways were analyzed using DAVID tool. The results showed that 22 miRNAs were differentially expressed between LGDHS and LKYDS (fold change >2.0 and *P* < 0.01). Circulating miR-583 and miR-663 were significantly higher (*P* < 0.001) in CHB patients with LGDHS than those with LKYDS and Ctrls. ROC curve analysis revealed that miR-583 and miR-663 were sensitive and specific enough to distinguish LGDHS from LKYDS. Pathway enrichment analysis indicated that 354 putative targets for miR-583 and 68 putative targets for miR-663 were mainly involved in Axon guidance, Neurotrophin, and MAPK signaling pathway. miR-583 and miR-663 may be potential markers for ZHENG differentiation in CHB.

## 1. Introduction 

ZHENG, which is also defined as traditional Chinese medicine (TCM) syndrome or pattern, is a basic concept of TCM theory [1]. TCM ZHENG is a profile of symptoms and signs as a series of clinical phenotypes, which reflects the essence of pathological changes at a certain stage in the process of disease occurrence and development. Therefore, ZHENG plays an important role in understanding the human homeostasis and guiding the applications of Chinese herbs and acupuncture. In TCM clinical practice, TCM diagnosis is a process of ZHENG differentiation in diseases. Moreover, the same disease can usually manifest in different ZHENGs, so patients with different ZHENG types should be treated by different rules and therapeutic regimen. 

Although TCM has been practiced effectively more than 3000 years, ZHENG differentiation is still argued. Because it is assessed by the clinical manifestations of patients through four diagnostic methods, including looking, listening and smelling, asking, and touching [2], ZHENG differentiation depended on clinical observation and TCM practitioner's experience, which would be subjective and unrepeatable. At present, a TCM ZHENG diagnosis is integrated with a biomedical diagnosis in clinical practice, and integrative medicine emerges as an optimal approach for achieving higher efficacy [3]. 

MicroRNAs (miRNAs) are evolutionarily conserved, small (typically −22 nt in size) regulatory RNA molecules that function to modulate the levels of specific targets. It is actively involved in a wide range of physiologic and pathologic processes [4, 5]. Recently, profiling of circulating miRNAs has been used in a number of studies to identify novel minimally invasive miRNA biomarkers. miRNAs are usually very stable in plasma and can be found in lipid or lipoprotein complexes [6], apoptotic bodies [7], microvesicles [8], or exosomes [9]. Many studies have shown that the levels of circulating miRNAs were altered significantly at different physiological stages and pathological conditions [10–20]. These circulating miRNAs are emerging as novel noninvasive biomarkers for the detection and prognosis of diseases [10–20].

Hepatitis B virus (HBV) infection is one of the major health problems in China [21]. Of the 350 million individuals worldwide infected with the hepatitis B virus, one-third of them reside in China [22]. HBV infection results in chronic hepatitis B (CHB) and patients with CHB exhibit high risk of developing liver cirrhosis and hepatocellular carcinoma [23]. TCM is widely used in the treatment of CHB and found to be effective in China [24–26]. Since the effective TCM treatment relies on the right ZHENG differentiation, the aim of this study is to investigate whether the circulating miRNAs as molecular biomarkers are able to differentiate ZHENGs in CHB.

## 2. Materials and Methods 

### 2.1. Subjects and Experiment Design

Sera collected from 59 CHB and 21 healthy donors (Ctrls) were included in this study. Samples were from 6 CHB patients with 2 kinds of ZHENG, which were 3 liver-gallbladder dampness-heat syndromes (LGDHS) including 2 male and 1 female and 3 liver-kidney yin deficiency syndromes (LKYDS) including 2 male and 1 female. The samples were detected by miRNA microarrays to obtain serum miRNA profiles. The miRNAs with altered levels were further verified using qRT-PCR with the sample from the remaining 53 CHB patients (32 LGDHS and 21 LKYDS) and 21 Ctrls. Serum samples of Ctrls were randomly selected from a collection of 120 individuals who had annual physical examination at Shanghai Shuguang Hospital in Shanghai, China. Samples of CHB were from patients seeking treatment in Shanghai Shuguang Hospital. The clinical parameters of these patients were shown in Table 1. This study was approved by the Institutional Review Board of Shanghai Shuguang Hospital. The selected 59 patients with CHB must be in accordance with the following criteria: (1) all patients were diagnosed according to both CHB and TCM ZHENGs and confirmed by chief physicians; (2) the diagnosis of CHB based on the guideline that defined by Chinese society of hepatology and Chinese society of infectious diseases in 2005 [27]; (3) the ZHENG differentiation referred to the viral hepatitis diagnostic standard that described by Internal Medicine Hepatopathy Committee of Chinese Traditional Medicine Association in December 1991 [28]. An informed consent was signed by each of the participants, and the study protocol conformed to the ethical guidelines of the Declaration of Helsinki (1964).

### 2.2. Serum Sample Collection and RNA Isolation

All serum samples were from freshly drawn blood and stored at −80°C. RNA in serum was isolated using a mirVana PARIS kit (Ambion, Austin, TX, USA) according to the manufacturer's protocol followed by the treatment of RNase-free DNase I (Promega, Madison, WI, USA) to eliminate DNA contamination. The concentration of RNAs was detected from serum that ranged 1.5–12 ng/*μ*L.

### 2.3. Serum miRNA Profiling and Data Analysis

The profiles of serum miRNAs of 6 CHB patients (3 LGDHS and 3 LKYDS) were generated using Agilent Human miRNA microarray V3 (Agilent Technologies, Santa Clara, CA, USA). The microarray chip is comprised of 2371 different probes for total 851 human miRNAs. One hundred nanograms of serum RNA was used for each array. The arrays were read using the Agilent microarray scanner and the data were extracted using Feature Extraction V10.7 (Agilent Technologies, CA, USA). All data were transformed to Log base 2. The differences between samples were calculated using unsupervised analysis (SAS system, Shanghai Biochip, Shanghai, China). Only those miRNAs with the fold difference >2.0 and *P*  value < 0.01 were considered significant.

### 2.4. Quantification of Serum miRNAs

QRT-PCR-based quantification of miRNAs (200 *μ*L of serum each participant) was performed with Bulge-Loop miRNA qPCR Primer Set (Ribobio, Guangzhou, China) and SYBR Green PCR Master Mixture (TOYOBO, LTD, Japan) according to the manufacturer's instructions using a Roche LightCycler 1.5 real-time PCR machine. The specificity of each PCR products was validated by performing melting curve analysis at the end of PCR cycles. All samples were analyzed in triplicates and the cycle threshold (Ct) was defined as the number of cycles required for the fluorescent signal to reach the threshold. The levels of miRNAs in serum were calculated using the formula 2^ΔCt^, where ΔCt = Ct of internal  reference − Ct of target miRNA. miR-24 has been reported to be consistently present in human serum [10]. Our pervious study showed that miR-24 presents stably in human serum [11]. Thus, miR-24 was selected as the internal control to standardize differentially presented serum miRNAs in RT-PCR quantification.

### 2.5. Target Prediction and Enrichment Information

The target genes of miRNA were predicted by TargetScan prediction software (http://www.targetscan.org/). Gene Ontology (GO) and pathways were analyzed using a DAVID online analysis tool (http://david.abcc.ncifcrf.gov/).

### 2.6. Statistical Analysis

The comparisons between groups were analyzed using Mann-Whitney *U* test, Pearson *𝒳* ^2^ test, Canonical correlation analysis, or Spearman correlation analysis whereas appropriate. Receiver-operator characteristic (ROC) curves were established to evaluate the difference in the levels of serum miRNAs among CHB with TCM ZHENGs (LGDHS, LKYDS) and Ctrls. All tests were two tailed and *P* < 0.05 was considered statistically significant.

## 3. Results 

### 3.1. Clinical Parameters of Study Population

The clinical parameters of CHB patients with TCM ZHENGs and healthy controls are shown in Table 1. Data including body mass index (BMI), alanine aminotransferase (ALT), aspartate aminotransferase (AST), *γ*-glutamyltransferase (GGT), alkaline phosphatase (ALP), total bilirubin (TBIL), Hepatitis B surface antigen (HBsAg), and HBV DNA were expressed as the mean ± SD. According to the statistical analysis, no clinical parameters were significantly different between LGDHS and LKYDS, indicating that the 2 TCM ZHENGs could not be differentiated by the general clinical parameters of CHB. 

### 3.2. Expression Profiling of Serum miRNA

To determine whether there was difference in serum miRNA profiles between LGDHS patients and LKYDS patients with CHB, we performed miRNA microarray with RNAs isolated from sera of 6 CHB patients (3 LGDHS and 3 LKYDS). Among total 851 miRNAs analyzed, 22 of them were differentially expressed between 3 LGDHS and 3 LKYDS by hierarchical clustering analysis (). Compared to LKYDS patients, 17 miRNAs were upregulated and 5 miRNAs were downregulated in LGDHS patients (fold  change > 2.0 and *P*  value < 0.01) (Table 2).

### 3.3. Serum miRNA of TCM ZHENGs

**Figure 1:**
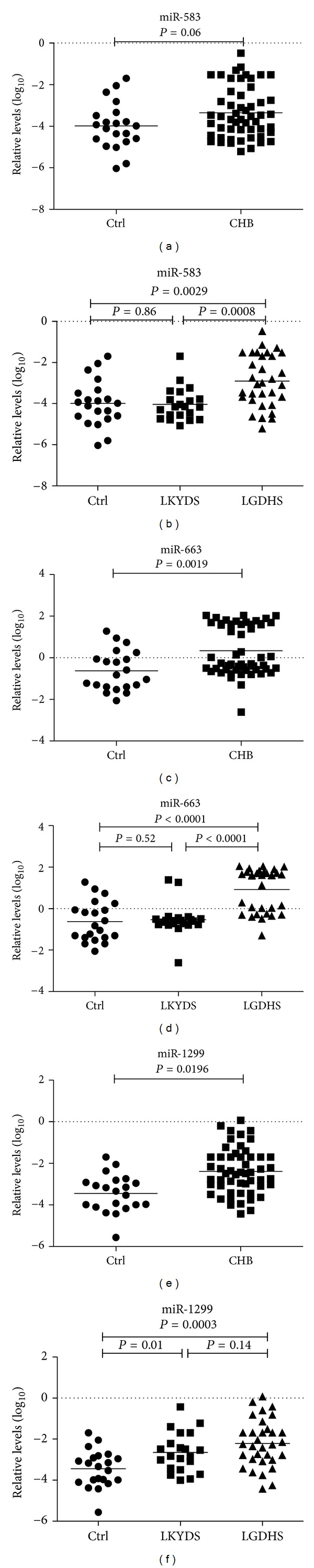
Serum levels of miRNAs in LGDHS, LKYDS, and Ctrls. The levels of serum miR-583 ((a), (b)), and mi-663 ((c), (d)), miR-1299 ((e), (f)) in CHB patients with LGDHS (*n* = 32), LKYDS (*n* = 21), and Ctrl (*n* = 21) were measured by qRT-PCR. The line at each group represents the median value of indicated miRNA. The values are normalized to miR-24 and shown in log_10_ scale at *y*-axis. (a), (c), (e): *P* values on the top are CHB versus Ctrl; (b), (d), (f): *P* values on the top are LGDHS versus Ctrl, on the left are LKYDS versus Ctrl, and on the right are LGDHS versus LKYDS.

Microarray analysis showed that greater numbers of up-regulated than down-regulated miRNAs are in LGDHS compared with LKYDS (Table 2). Among them, the median levels of serum miR-583, miR-663, miR-1299, miR-494, miR-140-3p and miR-936, and so forth were higher in 3 CHB patients with LGDHS than those in 3 CHB patients with LKYDS (Table 2). In order to validate these microarray-generated results, RNA was prepared from serum samples of another 53 CHB patients (32 LGDHS and 21 LKYDS) and subsequently subjected to qRT-PCR to measure the levels of miR-583, miR-663, and miR-1299 at random. The results showed that the levels of serum miR-583 were higher in all 53 CHB compared to Ctrls, but it was not statistically significant (*P* = 0.06) (Figure 1(a)). miR-583 was 18.35-fold higher in the sera of LGDHS than those of LKYDS (*P* = 0.0008), but there was no significant difference between LKYDS and Ctrls (*P* = 0.86) (Figure 1(b)). miR-663 was 9.61-fold higher in all 53 CHB comparing to Ctrls (*P* = 0.0019) (Figure 1(c)). MiR-663 was 15.64-fold higher in the sera of LGDHS than those of LKYDS (*P* < 0.0001), but no difference between LKYDS and Ctrls (*P* = 0.52) (Figure 1(d)). MiR-1299 was 2.93-fold higher in all 53 CHB comparing to Ctrls (*P* = 0.0196) (Figure 1(e)). MiR-1299 was higher in the sera of LGDHS and LKYDS than those of Ctrls (*P* = 0.01 and *P* < 0.0001, resp.) (Figure 1(f)). Compared to LKYDS, miR-1299 was higher in LGDHS, though it was not statistically significant (*P* = 0.14) (Figure 1(f)). These results demonstrated that a subset of miRNAs is differentially present in the sera of CHB patients with different ZHENGs.

### 3.4. Sensitivity and Specificity of Serum miRNAs for TCM Syndrome Classification

**Figure 2:**
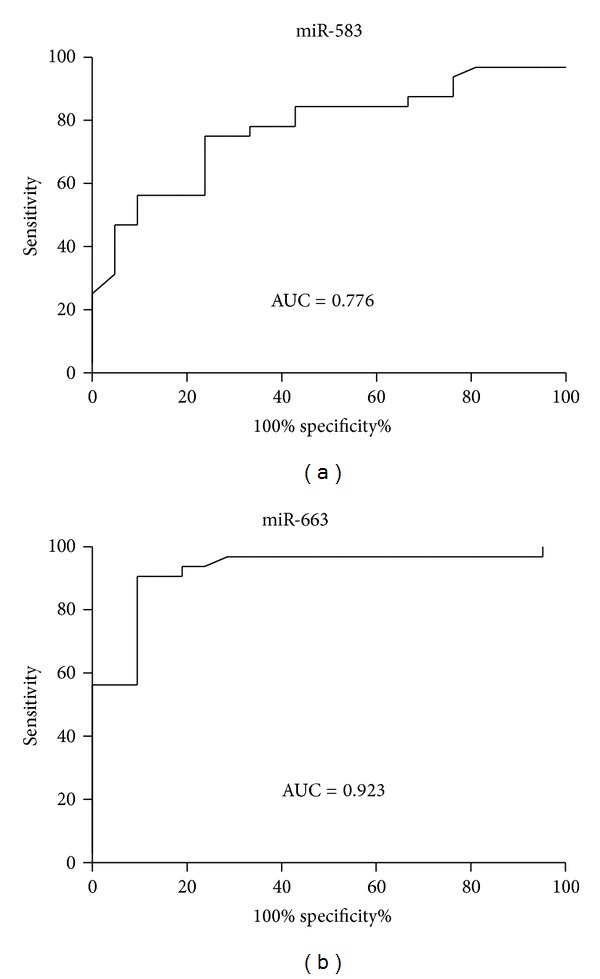
ROC curve for classification of two different TCM syndromes in CHB patients. It was generated combining the levels of serum miR-583 and miR-663. (a) ROC curve for classification of LGDHS and LKYDS. AUC (area under the curve) = 0.776. (b) ROC curve for classification of LGDHS and LKYDS. AUC = 0.923.

We compared the levels of serum miR-583 and miR-663 between LGDHS and LKYDS. ROC curve areas of miR-583 and miR-663 were 0.776 (95% CI: 0.65–0.90) and 0.923 (95% CI: 0.84–1.00) (Figures 2(a) and 2(b)). The sensitivity and the specificity, respectively, were 75% and 76.2%, 90.6% and 90.5% between LGDHS and LKYDS subjects. These results thus also show that the levels of miR-583 and miR-663 may distinguish LGDHS from LKYDS in CHB patients.

### 3.5. GO Terms and KEGG Pathway Annotation of the miRNA Targets

**Figure 3:**
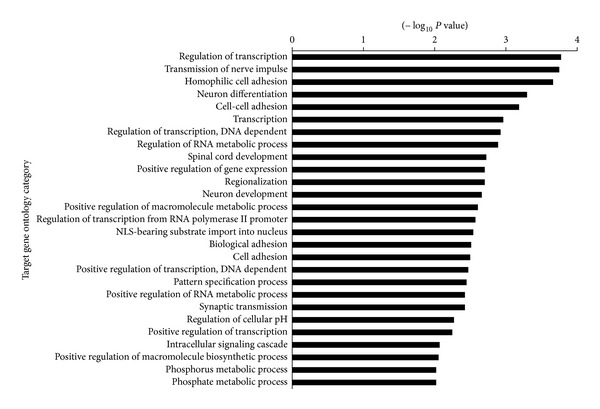
GO functional enrichment of miR-583 and miR-663 potential targets. GO enrichment score provided by David software as −Log_10_ *P* values (*P* < 0.01).

**Figure 4:**
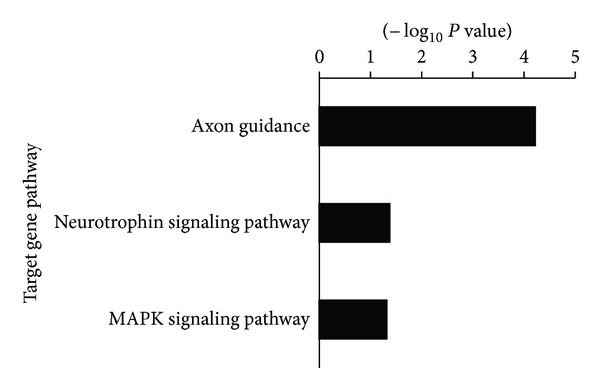
KEGG pathway annotation of miR-583 and miR-663 potential targets. Pathway enrichment score provided by David software as −Log_10_ *P*-values (*P* < 0.05).

To investigate the function of the miR-583 and miR-663, we predicted miRNA targets using TargetScan 6.2 database. There were 354 putative targets for miR-583 and 68 putative targets for miR-663. GO function and KEGG pathway enrichments were performed by mapping the predicted target genes, respectively. The results showed that 27 GO functions (*P* < 0.01) and 3 KEGG pathways (*P* < 0.05) are annotated. Therein, GO term annotation showed that regulation of transcription, cell adhesion, cellular physiological process, metabolism, and regulation of gene expression are the most significantly enriched GO terms (Figure 3). Pathway enrichment analysis indicated that putative targets for these miRNAs were mainly involved in Axon guidance, Neurotrophin, and MAPK signaling pathway (Figure 4).

## 4. Discussion

In TCM, the treatment of disease relied on ZHENG differentiation. There are several different ZHENGs in a disease, so the same disease should be treated by different therapeutic approaches [2]. However, ZHENG differentiation is usually based upon the physician's intuition and personal experience, which might result in different diagnosis from physician to physician and from clinic to clinic. So it is essential to find a scientific approach for the application of ZHENG differentiation in TCM.

LGDHS and LKYDS are 2 kinds of common TCM ZHENGs with different clinical phenotypes in CHB. In TCM, LKYDS is recognized to be the insufficiency of body fluid. It represents dryness in the throat and/or mouth, perspiration during sleep, tinnitus, dizziness, fatigue, insomnia, red tongue body with no coating on, and pulse that is thin, fine, or floating and empty; LGDHS is recognized to be the dampness and heat accumulated in liver and gallbladder. It represents epigastric or abdominal oppression, lack of appetite, heavy body weight, thirst with little or no desire to drink, abdominal pain, loose stools, nausea, vomiting, fever, headache, red tongue body with a yellow sticky coat, and/or slippery rapid pulse. There is still not a clear understanding of the biological validity and lack of biological markers for diagnosis of LGDHS and LKYDS in CHB.

miRNAs are a class of small noncoding RNAs that play an important role in the regulation of various biological processes such as cellular development, differentiation, proliferation, apoptosis, and metabolism through their interaction with cellular messenger RNAs [4]. Previous studies have shown that miRNAs not only circulated in the blood as a cell-free form, but also were acknowledged as readily accessible disease markers. It has been reported that the levels of miR-122, miR-133a, and miR-124 are specifically elevated in blood of patients with liver, muscle, and brain injury, respectively [12]. Recent studies suggest that circulating miRNAs may represent a new class of biomarkers for monitoring the progress of certain diseases [13–20], such as miR-9 as novel noninvasive molecular marker for traumatic spinal cord injury [14], miR-208a for early detection of acute myocardial infarction [15], and miR-146a/223 for the diagnosis of sepsis [16]. 

Our study has shown that miR-122, -638, -572, and -575 were presented at higher levels while miR-744 is at lower levels in the sera of patients with CHB and nonalcoholic steatohepatitis (NASH). The levels of these miRNAs not only correlated with liver pathological parameters, but also indicated the degree of liver injury than commonly used markers such as ALT and AST [11]. In this study, we further investigated the value of circulating miRNAs as potential biomarkers for TCM ZHENG differentiation in CHB. 

Our results showed that there are obviously different miRNA expression profiles and 22 of total 851 miRNAs are differentially expressed between LGDHS and LKYDS (fold change > 2.0 and *P*  value < 0.01). Therein, the levels of serum miR-583, -663, and -1299 were significantly altered in CHB patients with LGDHS and LKYDS in comparison with the healthy control (Table 2). Importantly, the alteration of these miRNAs correlated with well-established TCM ZHENG. MiR-583 or miR-663 was able to distinguish between LGDHS and LKYDS (Figures 1(a) and 1(d)). To observe the sensitivity and specificity of these miRNAs, ROC curve analysis was conducted to differentiate between LGDHS and LKYDS. The area under the ROC curve was 0.776 and 0.923, respectively (Figure 2). Although the investigation with greater number of samples may be necessary to fully validate our findings, our results suggested that the alteration of serum miR-583 and/or miR-663 may be a potential molecular biomarker for predicting ZHENG differentiation in CHB. Moreover, these results also supported the hypothesis that serum miRNA profile may be used to the ZHENG differentiation in CHB. 

In addition, it is unclear that whether serum miR-583 and miR-663 are liver derived or originated from immune cells involved in the antiviral responses that accompany the development of liver diseases. Using a bioinformatics approach, we analyzed potential targets of the miR-583 and miR-663. GO enrichment analysis indicated that the 422 putative targets of these miRNAs were mainly involved in regulation of transcription, cell adhesion, metabolic process, and so forth (Figure 3). Pathway enrichment analysis indicated that putative targets for these miRNAs were mainly involved in Axon guidance, Neurotrophin, and MAPK signaling pathway (Figure 4). It was reported that Axon guidance, Neurotrophin, and MAPK signaling pathway play important roles in the pathophysiology of liver, which are involved in the development of hepatitis [29], liver fibrosis [30, 31], and HCC [32]. It indicates that the miR-583 and miR-663 have some important functions by regulating these pathways in liver cells. Further study will investigate the expression of miR-583 and miR-663 in a variety of clinical liver biopsies and related cell lines. It might help to elucidate the biological role in the progression of chronic liver diseases. 

This study demonstrated that miR-583 and miR-638 were presented at higher levels in the sera of LGDHS than those of LKYDS in CHB patients. It provided an evidence to objectively differentiate TCM ZHENGs in CHB. However, there also exist some limitations in the study, such as the small amount of study population and lack of identification of miRNA function in the liver, which would be researched in future study.

## Figures and Tables

**Table 1:** Clinical parameters of participants in validation.

Parameters	CHB with LGDHS	CHB with LKYDS	Ctrl
Individuals (*n*)	32	21	21
Male	26	15	15
Female	5	6	6
Age (years)	37.9 ± 12.8	37.4 ± 13.7	35.23 ± 11.4
ALT (IU/L)	73.1 (10–412)	82.8 (19–354)	22.3 (14–43)
AST (IU/L)	53.3 (25–207)	60.0 (21–225)	22.0 (16–49)
GGT (IU/L)	53.5 (11–180)	46.6 (16–285)	20.8 (12–33)
ALP (IU/L)	89.3 (51–185)	84.6 (44–156)	62.3 (42–96)
TBIL (*μ*M/L)	16.2 (7.6–38.5)	17.4 (7.6–27.2)	16.3 (6.9–27.1)
HBV DNA	6079004 (0–94800000)	6706693 (0–62270000)	0
HBV status (*n*)			
HBsAg+	32	21	0
HBsAg−	0	0	21

Ages are given as mean ± S.D.; values of alanine aminotransferase (ALT), aspartate aminotransferase (AST), Gamma-glutamyltransferase (GGT), alkaline phosphatase (ALP), total bilirubin (TBIL) and HBV DNA were given as medians (range). LGDHS: liver-gallbladder dampness-heat syndrome; LKYDS: liver-kidney yin deficiency syndrome; Ctrl: healthy donor; HBsAg: hepatitis B surface antigen; HBV: hepatitis B virus.

**Table 2:** Differentially expressed miRNAs in CHB patients with LGDHS and LKYDS.

miRNA	Fold change (LGDHS/LKYDS)	*P* values
hsa-miR-494	13.66	0.0002
hsa-miR-140-3p	13.01	0.0000
***hsa-miR-663 ***	12.81	0.0028
hsa-miR-187*	11.64	0.0002
hsa-miR-936	11.43	0.0002
hsa-miR-361-5p	10.50	0.0055
hsa-miR-148a	9.49	0.0016
hsa-miR-149*	9.44	0.0018
***hsa-miR-1299 ***	8.83	0.0001
hsa-miR-708	8.81	0.0068
***hsa-miR-583 ***	8.22	0.0006
hsa-miR-760	7.47	0.0023
hsa-miR-184	7.42	0.0008
hsa-miR-30a	5.48	0.0015
hsa-miR-30e	4.88	0.0029
hsa-miR-887	4.44	0.0061
hsa-miR-345	4.07	0.0031
hsa-miR-875-5p	0.15	0.0004
hsa-miR-15b*	0.13	0.0000
hsa-miR-329	0.06	0.0011
hsa-miR-369-5p	0.03	0.0043
hsa-miR-211	0.02	0.0064

*miRNA cloning studies sometimes identify two ~22 nt sequence miRNAs which originate from the same predicted precursor. When the relative abundancies clearly indicate the predominantly expressed miRNA, the mature sequences are assigned names of the form miRNA (the predominant product) and miRNA* (from the opposite arm of the precursor). For example, miR-123 and miR-123* would share a pre-miRNA hairpin, but more miR-123 would be found in the cell. This distinction was also made with “s” (sense) and “as” (antisense) previously.
